# Understanding Violence Against Women in the Caribbean Through an Exploration of Men's Perspectives

**DOI:** 10.1177/10778012221104845

**Published:** 2022-08-09

**Authors:** Debra D. Joseph, Adele D Jones

**Affiliations:** 1 41633 The University of the West Indies, Cavehill, Barbados; 2 The University of Huddersfield, Huddersfield, UK

**Keywords:** domestic violence, gender-based violence, men, Caribbean

## Abstract

Qualitative research with 60 males (16–80 years) from two Caribbean countries was carried out to explore men’s perspectives on domestic violence (DV). An inductive latent/thematic approach to data analysis supported by analytic software led to five key domains being identified: (1) meanings of violence; (2) patrinormative culture; (3) normalization of violence; (4) male victimization; and (5) blame attribution and empathy. Patriarchal values, together with childhood exposure to violence, were found to reduce empathic capacity and contribute to the normalization of DV. In addition, the minimization of male victimization and the lack of behavior-change support services for men were identified as contributory factors.

## Introduction

Gender-based violence (GBV) is a widespread public health issue limiting the lives and livelihoods not only of women, but also of sexual minorities and transgender people (Heise & Garcia-Moreno, 2002; Nakray, 2013; Tjaden & Thoennes, 2000). The most common form of GBV is domestic violence (DV) (also referred to as intimate partner violence), and the World Health Organization (WHO, 2013) estimates that 35% of women globally are subjected to physical and/or sexual violence and around 38% are killed by an intimate partner. The Pan American Health Organization highlights that although women can perpetrate violence against intimate male partners, in most instances DV is perpetrated by men against women (n.d.). This, of course, excludes same-sex relationships. It should be noted, however, that in a study of gay men and lesbians, Murray et al. (2007) found that DV in intimate relationships was as high or higher than rates found in heterosexual relationships.

In the Caribbean, violence against women is acknowledged as being extensive across the region (Bott et al., 2012; Contreras et al., 2010; IDB, 2018; Quamina-Aiyejina & Brathwaite, 2005). Sexual coercion and abuse are reportedly widespread (Blum et al., 2003; Jones & Trotman Jemmott, 2009) and five of the top 20 recorded rape rates for 2019 were in the Caribbean: Grenada recorded 30.6 per 100,000; St. Kitts and Nevis, 28.6; St Vincent and the Grenadines 25.6; Barbados 24.9, and Jamaica 24.4 (World Population Review, 2019). In a survey of 1,079 women in Trinidad and Tobago, 30% of ever-partnered women had experienced physical and/or sexual violence and 7% of all respondents said they had been forced into sexual intercourse by a non partner (Inter-American Development Bank [IADB], 2018). As elsewhere, the most prevalent form of violence against women in the Caribbean is that which occurs within the context of intimate relationships (Bott et al., 2012), commonly referred to in the region as DV. Across the Caribbean, legislation exists which criminalizes physical and sexual assault and many countries now have specific laws targeting DV. However, the implementation and regulation of DV laws are largely ineffective and violence against women remains deeply entrenched. There are several reasons for this including the lack of joint working across social and political sectors, inadequate budgetary allocations, poor harmonization of law with policy, and limitations of data gathering and monitoring systems. These challenges are underpinned by patriarchal values that seem resistant to the prioritization and promotion of laws designed to protect women's rights and freedoms (Lacey et al., 2019; UNDP and UN Women, 2017).

Few scholars have investigated the determinants of DV in the Caribbean and explanatory theories have so far proved inadequate in capturing the complex meld of factors involved. As Le Franc et al. (2008) argue, reducing violence against women in the Caribbean requires an examination of the meanings of violence, not only among those who are its victims but also among men, including those who perpetrate such violence. The literature from the English-speaking Caribbean, where this study takes place, suggests that it may be difficult to disentangle violence against women from the region's deep historical, enslaved, and colonized roots and the systematic emasculation of Caribbean men that ensued. If violence is examined not only in terms of individual psychopathology, but by situating it within its social, economic, political, and historical context (Mohatt et al., 2014; Tawil, 2013) it seems instructive to acknowledge that consequent on slavery and colonization, control and domination were the founding principles of Caribbean societies (Ashcroft et al*.*, 2013) with status and privilege being reserved for men. From these histories of learning, gendered positioning became enculturated and though Caribbean women have long owned legal rights for sexual, economic, and political freedom, the region's historical legacy means that women are sometimes still treated as their husband’s property and men continue to regard the threat of emasculation as justification for violence (Lacey et al., 2019). Edmonds (2012) analyzed the spike in the prevalence of DV homicides in the Caribbean following the global economic downturn which took hold in 2011 and concluded that a contributory factor was the psychosocial challenges men faced in reestablishing their position of dominance following the loss of employment, status, and income. He referred to this phenomenon as a “crisis of masculinity.” DV in the Caribbean, as elsewhere, is perpetrated primarily by men (Bott et al., 2012; Imbusch, 2011) yet little attention has been given to male views on its contributory factors. However, a qualitative study in two Caribbean countries involving 60 male participants aged 18–80 years, supported Edmonds’s earlier observations and reported that many men believed their masculinity was threatened if they were insulted by their partner, and this, in turn, was perceived as a motive for violent behavior (Jones et al., 2017). A deeper exploration of men's perspectives therefore seems crucial to increasing understanding of the roots of violence against women, its trajectories, and the factors that sustain it.

Responding to the need for new insights into this long-standing problem, this study aimed to explore factors implicated in men's individual and collective perceptions about gender, masculinities, women, and violence. In the sections that follow, we first provide a description of the setting for the study; we then describe the methods and then the findings. We discuss the findings in relation to key literature relating to the major themes and we conclude with our observations concerning the policy and practice implications of the research.

### Setting

The study took place in two English-speaking Caribbean countries, Barbados and Grenada; countries that have a shared history of slavery and colonialism. Both countries are in the high human development category (reflecting universal access to education, long-life expectancy—averaging about 75.5 years), and commensurate rates of per capita income. This is important because low rates of social development have been associated with societal attitudes that justify DV and have been said to predict rates of perpetration and victimization (Sardinha & Catalán, 2018). In 2017, Barbados had a Human Development Index (HDI) of 0.800 while the value for Grenada was 0.772 (ranking them 58th and 75th, respectively, out of 189 countries) (UNDP, 2018b). Notwithstanding their HDI rankings and Barbados’ status as having one of the lowest gender-pay disparities in the world (UN Women, 2019), gender inequality remains entrenched in both countries. Patriarchal values prevail in both Barbados and Grenada and gender stereotypes and structural barriers to equality mean that women are often forced into subordinate positions (Drakes & Perks, 2013; UN Women, 2018). Women experience poverty and unemployment at higher rates than men with the greatest burden being borne by single female-headed households; in Barbados 47.5% and in Grenada 41% of all households are headed by women (Caribbean Development Bank, 2016).

## Method

This article is based on a subset of data gathered between 2016 and 2018 as part of a study by the None in Three Centre for the Global Prevention of Gender-based Violence which aimed to explore the social etiology of DV in Barbados and Grenada. The aim of the current study was to explore male perspectives on DV. Although it is not assumed that Barbados and Grenada share the same cultural norms, it *is* widely acknowledged that there are regional commonalities in the manifestations of patriarchal assumptions and the embeddedness of structural gender inequalities (Quamina-Aiyejina & Brathwaite, 2005) that apply to both countries and the data have therefore been merged.

### Participants

The study consisted of a secondary analysis of transcripts from focus groups with men. Convenience, purposive, and nonprobability sampling techniques were used to identify a diverse sample of single and ever-partnered males (16–80 years) from three socioeconomic groups: unemployed, manual/low-skilled workers, and professional/high skilled and including males who had been convicted of a DV-related offense as well as males from the general public. The study utilized a cross-sectional qualitative design—data collected at a one-time point through guided focus-group discussions with purposively selected participants; eight focus groups were held, four in each country. The objectives were to ascertain the views of men concerning DV.

A total of 60 men (age 26 + ) and young adults (16–25) took part, 14 men and nine young adults from Barbados and 23 men and 14 young adults from Grenada (information on individual demographic characteristics was not recorded at the request of participants). Among the sample, 11 of the participants had been convicted of DV-related offenses, four men and three young adults from Barbados and two men and two young adults from Grenada. Offense details were not sought but all of the males who had perpetrated DV-related offenses had taken part in a court-mandated treatment program.

Prompt questions included: how men define DV; views on prevalence, causes, and effects; how living with DV affected them; situations/circumstances that contribute to men being violent; views about the help that men need. Ethical approval for the study was granted by the University of Huddersfield, School of Human and Health Sciences Ethics and Integrity Committee. Briefing information emphasized the voluntary nature of participation and the right to discontinue at any time. Participants gave informed consent and focus group participants selected pseudonyms to protect their anonymity. All focus groups were conducted in private settings, and the need for confidentiality was stressed.

### Analysis

Focus groups were digitally recorded, recordings transcribed and an inductive latent/thematic approach applied to data analysis. The NVivo software program (v10) (QSR International, 2012) was used for coding. Key themes were collaboratively identified by both authors to generate a codebook to guide the iterative evaluation of all data. Text searches were carried out for content validity, to identify negative cases, and to check for coding density. Given the nature of qualitative research, lexical searches cannot be reduced to their frequency counts and phrases were therefore analyzed in the context of the surrounding text. A constant comparison between cases enriched analysis by pointing to negative cases where men described developing nonviolent tendencies despite being immersed in subcultures of violence acceptance. More complex matrix queries based on node attributes highlighted segments of the transcripts for further thematic analysis. The authors jointly agreed on the selection of quotations to best illustrate the themes.

## Findings

Of the participants, 18% (*n* = 11) were men and young adults who had been convicted of DV-related offenses, while the majority (*n* = 49) were men and young adults without any DV-related convictions. The intention, in combining both groups, was to explore differences in perspectives depending on whether participants had engaged in a psychosocial treatment program or not (all the offenders had). Two important differences emerged. Males who had participated in a treatment program demonstrated greater awareness of the situation of women and were less likely to blame the victims of DV than the participants from the general population. However, given the small number of offender participants (*n* = 11) this finding should be regarded as tentative. Concerning age-related differences, older men (26 + ) tended to subscribe to more traditional gender stereotypes than young adults (18–25) and young adults suggested different coping strategies, such as the use of marijuana rather than alcohol. The younger participants also argued for youth-led counseling interventions in addition to the men-to-men approaches advocated by older males. Beyond these points, there were no other notable differences across the groups and the data were subsequently merged for further analysis.

In order to display the conceptual range of findings, the authors developed a taxonomy of responses based on the full list of nodes created for the NVivo analysis (nodes for the young adult sample [16–25 years] and the adult male sample [>25 years] were integrated). Using a deductive approach, this involved moving between nodes and the original transcripts to identify and code units of text and references based on the taxonomy. Items that shared a semantic relationship were synthesized to produce five key domains: (1) meanings of violence; (2) patrinormative culture; (3) normalization of violence; (4) male victimization, and (5) blame attribution and empathy (see Table 1).

**Table 1.** Male Perspectives on Domestic Violence (DV): Key Domains.

NVivo Nodes	Direct Comments	Other References	Key Domains
Defining DV	60	68	Meanings of violence
Prevalence, causes, and effects	19	99	
How common DV is	53	90	
Factors that contribute to violence	53	93	

Escalatory factors	19	40	Patrinormative culture
Factors that contribute to violence	13	93	
Why men hit women	37	78	
Challenges, strengths, and strategies	55	149	
Pressures	16	86	
Impact of these situations on men	12	97	

Effects of DV on families	19	72	Normalization of violence
Violence in society	42	32	
Effects of violence on boys/youth	40	14	
Escalatory factors	19	40	
Factors that contribute to violence	33	93	

Male victimization	18	40	Male victimization
Effects of violence on boys/youth	40	14	
What help boys/men need	14	44	
What help available	7	12	
Coping strategies used	16	86	

Views about the role of women	12	97	Blame attribution and empathy
Effects of violence on boys/youth	40	14	
Escalatory factors	29	40	
Factors that contribute to violence	33	93	
Would never hit a woman	7	78	

The use of focus groups meant that it was not always possible to attribute responses to individual men or to determine precisely how many participants shared a view being expressed and for this reason, in presenting the findings, phrases indicating consensus rather than actual numbers are sometimes used. However, the use of NVivo software enabled the authors to accurately determine the frequency of recurring responses and in many instances, to track these to the participants expressing them; where this was possible, the numbers or percentages of respondents are given.

### Meanings of Violence

Out of 60 participants, 98.3% (*n* = 59) did not identify sexual coercion as a form of DV and referred only to physical and psychological acts of harm. Only two respondents mentioned economic control as a form of abuse although several men thought that women's lack of economic power made them vulnerable to abuse. More than half of the interviewees questioned the ways in which DV had been defined by policymakers and their comments suggested that dominant definitions had generated a polarized view of the problem resulting in the minimization of some forms of DV. Broader conceptualizations were called for which include all forms of abuse and interpersonal violence, including violence perpetrated by women and violence against and between children.

I know young men who were abusing at 13,14 years old, but we don't want to admit that you know because you see this sibling war, boys beat their sisters and vice versa, that is abuse, that is domestic violence.

Discussants talked extensively about the impact on boys of exposure to multiple forms of violence including child abuse, peer/gang violence, school violence, and violence perpetrated through the media.

These young men and these young boys are suffering in untold ways due to this violence that takes place in the home and the kind of abuse that they see.

Aggression was viewed by about three-quarters of the participants as a “natural” trait that was generally kept in check but triggers such as stressors related to unemployment and poverty and psychological responses to jealousy and insecurity were said to “cause a man to lose control” and could lead to him being violent. A key trigger said to contribute to the loss of control and violent behaviors was alcohol. However, it was not alcohol alone that was seen to be the problem, but the abuse of alcohol in a context in which male violence was socially sanctioned.

…there were three or four guys in my village, that you could guarantee that every Friday evening, that them three women buss up [their three wives were beaten up]. Every Friday evening like clockwork. You could set your clock on the wall by two of them, because you know that he would come in about half past eight drunk from the shop, and licks [punches or slaps], you don't have to ask no questions.

I think it is mostly influenced by alcohol, rum. I never see a sober man beat a woman yet, but as soon as they influence by alcohol they beat the woman.

### Patrinormative Culture

This domain yielded the largest number of references (see Table 1) and indicated that men were expected to behave in dominant and aggressive ways and that women should not be treated as equals. Toughness, aggression, and controlling behavior were identified as normative markers of being male. Boys were said to be discouraged from “being emotional,” and many interviewees had learned from childhood that men should repress their feelings. Most men (*n* = 52) expressed views that indicated they had grown up with cultural norms that meant boys were not encouraged or expected to recognize, express, or manage their feelings and this led to an inability to regulate emotions.

I feel man should behave a little more better with they self. Learn how to control their anger, but eh have a lot a man they just hyper, any little thing does just trigger them off; … Them have to learn how to control their anger.

Participants suggested that growing up in the midst of violence influenced the kind of psychological tools developed for responding to conflict and when tensions boiled over because males were expected to be in control, they often behaved aggressively.

They [boys] are encouraged to behave in violent ways, you know. When they speak to their peers they would be encouraged in fact to continue [being violent]. A lot of times, um men, they male friends would actually support them [in being violent] and encourage them to be a man because a lot of times domestic violence is linked to being male, being a real man.

This is all that I know … this is what my mother and my aunts would have told me that I ain't gonna end up anything better, and therefore “well, hey,” self-fulfilling prophecy I going to become the very thing they claim I to be.

Society itself has influenced men that they are in charge. Society itself does not teach men that women should be of an equal partner with them and … because of the way they grow, men believe they are in control and they dictate everything that a woman should and should not do.

Set alongside these findings, 25% (*n* = 15) of the participants said they were opposed to all forms of violence and even if they had grown up with these cultural mores, rejected the notion that violence was a part of their identity as males. These men spoke about the strategies that had helped them in dealing with relationship conflict; religion and the influence of positive male role models being the most common. However, men who had witnessed DV as children or who had been subjected to abuse themselves often drew on those same repertoires of violence and male dominance even as they demonstrated their opposition to it.

I’ve heard a lot of young men say “you see me, I’m not gonna beat my partner like how my father did to my mother” and there are a lot of young fellas now that have a different attitude and they try, they try to close the gap between them, they and their partners. But some partners are very rebellious and resilient.

…only the other day … I saw a man, punching the hell, the living daylights out a lady …and I was 2 ½ seconds from rearranging his face … just when I folded my fist ‘cos I was gotta hit him hard, I know if I had hit him actually I probably would have killed him … and a lady jump in between us two and perhaps that's the reason why I’m here talking to you today….

If I see a man hit a woman, I get extraordinarily cruel [angry] and I would actually do far more damage.… So for me, seeing violence as a boy, I decided that I would never do it or that I would never tolerate it being done in my presence, that's what it did for me.

### Normalization of Violence

About three-quarters of the participants described DV as something they had witnessed or been exposed to on a regular basis from early childhood—“Where we’re from, domestic violence is a normal thing, you see it every day and you just walk your way.” Some participants suggested it was so common as to be unremarkable—“It's just human nature to us,” while others said that despite its prevalence, witnessing DV was traumatic.

The gentleman who lived next door to my grandmother's house, pretty much on a daily basis used to beat the hell out of his lady. On more than one occasion she was either half naked or fully naked and as a boy, 10–11 years old I saw that.

When you look at it, the violence is generational, can't get away from it, it is generational…. You go back through some of the family histories, their grandfather and the great grandfather, was an abuser and it has come forward to now.

Many men (*n* = 52) expressed comments that indicated that behaving aggressively was a default position, a choice often made unthinkingly because in their communities, children, and boys, in particular, were socialized into accepting violence as normal.

“…wuh monkey see, monkey do” [referring to mimicking an action without understanding or thinking about the associated consequences].

If we are talking about young minds, young impressionable minds and if … these children are seeing this violence and abuse being perpetrated in their presence, now what do you think they will learn? That is the question I will ask because you could only learn what you are taught…. So what the children are seeing they will manifest later on in life.

### Male Victimization

Participants acknowledged that DV was largely perpetrated by men against women but were concerned that violence against men was often minimized or trivialized.

Sir, a girl and her mother beat a man badly … but, there is no recourse … I’m yet to see it happen. I just hoping that with this new protection order act [new domestic violence law] that it respond also when men call police. But before men were being laughed at and the police never really do nothing.

Emotional abuse, perpetrated by both males and females was highlighted as a major problem, and respondents believed there needed to be more emphasis placed on this aspect of DV.

There's no more cruel abuse than the psychological abuse a woman portrays and puts on a man, no more cruel than that! … I’ve seen men who will sit and take the verbal crap dished out by women and don't say a word. Men cut, I mean these men are emasculated in a way that you can't believe … because it [physical violence] is the most obvious one to see, the one that is manifested most, but the psychological abuse, which is the most terrible form of abuse, nobody dealing with this….

I see my mum cry so many times just for words; my dad had some words that I don't think you’d find none of them in the dictionary, he had devise a dictionary that you wouldn't wanna know, he doesn't used to stop.

All participants believed that it was crucial to recognize the male not only as perpetrator but also victim and the female as not only victim but perpetrator too. Although the men acknowledged that gender inequalities meant that women were more likely to be victims of DV, they also believed that gender inequalities meant that abuse against males was not taken seriously and that dismissive responses actively contribute to the normalization of violence.

It is still considered that if a woman hit a man, he can tek [take] it but when a man hit a woman she can't. That is still very much ingrained in our societal ethos, our thinking and that … is where the problem is coming.

A woman will tell a man today, “you ain't know you want stabbing up?” [you should be stabbed] and the police … [will say], “what is the problem?… Man, relax yuhself bossman…” [don't take it seriously]. But reversal now, “man, he got to leff [leave] the place, ‘cos this is a threat”.

Men decried the lack of services for male victims and also, for those men who had subsequently become the perpetrators of violence as a consequence of their own socialization into violence. Eleven of the participants had participated in court-mandated behavioral programs which had helped in changing behaviors, however, these were only available to offenders convicted of DV and most of the participants believed that such programs should be more widely available.

Me, before time I didn't know how to avoid violent free and thing [avoid being violent] until after I come to that programme from the court, and not to say thing but it teach me a lot.

Most participants indicated that they felt excluded from gender policies and programs but had many suggestions for tackling DV, including counseling designed specifically for males and accessible at the community level both as a preventative measure and also to help in conflict resolution and secondly, male-to-male interventions to help men and boys reflect on their behaviors and attitudes.

### Blame Attribution and Empathy

A dominant theme expressed by at least a third of the participants was the belief that women are to blame for DV because of their “provocative” behavior. Examples of perceived provocation included “disrespecting the man,” “infidelity,” and not fulfilling expected roles appropriately. Key discourses underpinning this finding were two-fold: a belief in aggression being an “innate” male tendency which was used to justify the lack of self-control in acts of violence and secondly, a belief that a woman should “know her place” and behave accordingly in order to avert violence. Some men believed that women's economic advancement generated insecurity among males and argued that this could lead to frustration which in turn might lead to violence.

…women are working for more money than men and the women are telling their children “I don't want two idiots in the house,” referring to the father. That's an insult! Alright? “I don't want you like your old foolish father.” And so therefore they abusing, and therefore that could cause a man to retaliate because you have your pride and ego.

Blaming women was also associated with a lack of empathy for female victims and the minimization of DV: “There might be situations where a man might feel that um the woman deserve the violence.”

## Discussion

One of the most consistent findings in the literature is that being socialized in a home where DV or child abuse is perpetrated increases the likelihood of victimization or battering in adulthood (Guille, 2004; McIntosh, 2002). This was a dominant theme to emerge from the research and participants were strongly of the view that one of the primary reasons DV is high in the Caribbean is that boys learn to behave in violent ways because they witness it and experience it regularly at home. This is supported by Boduszek et al. (2017), who conducted a survey of over 600 boys from two Caribbean countries which revealed high rates of victimization among boys, both as secondary victims through the witnessing of DV and also as the primary targets of physical abuse. Almost a quarter of the boys (*n* = 136) were regularly exposed to DV in the home while 40.6% had been directly subjected to physical violence (Boduszek et al., 2017). These findings confirm other Caribbean research on the prevalence of boys’ exposure to violence in the family (UNICEF, 2015a, 2015b). Men failed to recognize sexual violence or abuse as a significant source of harm and 59 out of the 60 participants did not include sexual violence or sexual coercion in defining DV. Jones and Trotman Jemmott (2009), in a mixed-methods study, investigated attitudes to child sexual abuse in the Caribbean among over 1,400 adults and identified patriarchal sexual entitlement as a dominant factor in the minimization of sexual abuse. They concluded that in contexts in which concepts of masculinity intersect with gender inequalities and sexual cultures that preserve male privilege, sexual abuse, and coercion may not be conceptualized as such. This may help to explain why the men in the current study did not view DV as also including sexual violence and why there persists a general perception among some males in the Caribbean that rape within marriage is not rape (Elvy, 2015).

Many of the participants drew on repertoires of misogyny to excuse male violence. Negative attitudes to females have long been associated with gender inequality and it is argued that the family is the primary vehicle through which such attitudes are transmitted and where patterns of violence become established and rationalizations for gender-based violence formulated (Barnett et al., 2005; McCue, 2008). The current research found that juxtaposed with negative views about females were positive associations of masculinity with traits of domination and control. More than half of the participants suggested that a woman's economic and educational advancement could be considered a form of emasculation, especially if her male partner was unable to meet the material needs of the household. In these circumstances, DV was considered more likely and was even justified; in such instances, victims were regarded as the arbiters of their own abuse. This perhaps explains why only two out of 60 participants identified financial domination as a form of DV.

Despite being exposed to similar levels of family and community violence as their peers, many men rejected culturally inscribed patrinormative values that require them to be the holders of power and control within a relationship. And neither were they willing to view the woman's status as derivative or subservient. Instead, these men had incorporated values of nurturance, compassion, and nonviolence into their belief systems. A common factor in these cases was the existence within their lives, of other nonviolent men and also, older men in the community who did not dismiss their concerns and relationship conflicts, but who guided and supported them. Indeed, they recommended that male-to-male interventions, through which men challenge anti-woman discourses and help to develop nonviolent problem-solving strategies could be an important strategy for addressing DV. Exploring the value of programs for men who commit DV is beyond the scope of this article nevertheless it appears that the most successful batterer intervention programs are those that, in addition to prioritizing victim safety, hold men accountable and enable the reflexive deconstruction of masculine constructs that reify control and domination (Gondolf, 2012; Heise, 2011).

An important factor affecting the reproduction of violence is its normalization and the social processes that contribute to its acceptance. These processes include the social construction of masculinized identities which, developed in the context of the family and affirmed by peers and others, shape the attitudes, perspectives, and behavior of young men (Wiltshire, 2012). In her study of youth, masculinities, and gender in five Caribbean countries, Wiltshire found that schooled in cultural contexts in which aggression and dominance are common signifiers of “manhood,” young males tended to resort to violence as a first response to conflict. Although most of the 518 young males in Wiltshire's survey said they rejected violence against women, many thought it was necessary to “discipline” females in certain circumstances, such as when they felt insecure or needed to display power (2012, p. 4). The alignment of hegemonic masculinities with controlling behaviors is supported by several Caribbean scholars. Reddock (2004), for example, argued that due to the high social value given to dominant perceptions of what it means to be a man, males are often under intense pressure to prove their eligibility for recognition and this can lead to the exercise of control, especially over females. Wiltshire (2012) surmises that boys in the Caribbean approach relationships with the belief that they are expected to be dominant. The current research supports this and shows that DV is reinforced through wider sociocultural values that position dominance and aggression as acceptable, even expected masculine traits. As Gibbons (2015, p. 12) contends, violence prevention strategies need to engage men, not as the holders of power but as “members of families, husbands, fathers and partners who need to feel comfortable in their roles without resorting to violence to protect roles or problem-solve.” In order to disrupt the association of masculinity with violence and aggression, it is important to recognize that the “masculine stereotype damages the development of boys” emotional intelligence and their capacity to exercise self-discipline and self-control (Wiltshire, 2012, p. 4) and early interventions focused on young males is therefore crucial.

In environments in which interpersonal violence is considered a regular, everyday aspect of life, females can also become socialized toward its acceptance, and not only as victims. A strong theme from the current research was that female-perpetrated DV was not taken seriously by professionals and that views about male dominance made it difficult for men to report experiences of victimhood or to obtain support. Notwithstanding the cultural challenges male victims faced in disclosing abuse perpetrated by females, they believed the disparity in professional responses reflected gender priorities skewed against men. Although the reported rates of DV against males may be low, women *do* commit acts of violence against their partners. Le Franc et al. (2008) conducted a survey of DV among 3,401 young people (15–30 years) in Barbados, Jamaica, and Trinidad and Tobago. Approximately three-quarters of all respondents (ranging from 63.1% to 72.5% for men and from 65.1% to 83.1% for women) reported being a victim of some form of violence, most commonly perpetrated by a partner (59.0% for male victims and 66.7% for female victims). Overall women were 20% more likely than men to be victims of any violence and four times more likely than men to be victims of sexual coercion; however, around 50% of all respondents reported being victims of physical violence and with the exception of sexual coercion there were no significant differences by gender for the three countries as a whole. Furthermore, men reported similar rates of emotional abuse as women (up to 70%). These results concur with the current research in which some men described verbal cruelty as one of the contributing factors to antagonistic relationships. It is important to note, however, that surveys do not take account of gendered socialization processes and differential lived experiences which can lead to the minimization of aggression in reporting by men and women being more likely to describe their own actions in terms of violence (Baxendale et al., 2012). Debowska Boduszek, Jones, et al. (2018) suggest that given violence against women is widespread, many Caribbean women may fail to construe the way they are treated as abusive and this can affect reporting rates while other scholars have shown that much female perpetrated violence is retaliatory or in self-defense (Saunders, 2002). Nevertheless, the current research supports the conclusions reached by Le Franc et al. (2008) that both men and women can contribute to hostile relationship dynamics. Le Franc et al. describe this as “the existence of a universal culture of adversarial relationships, in a global environment permissive of the expression of violent solutions” (p. 417). Violence as a feature of interpersonal relations can become embedded within family and community life and may be one of the primary means by which children learn to emulate adversarial rather than non violent conflict resolution skills (Jones et al., 2016). In this regard, the current study suggests women as well as men may be implicated.

A key finding of the current study was the attribution of blame to victims and the failure of perpetrators to take responsibility for their actions and related to this, the lack of empathy. Many participants offered up victim-blaming narratives to justify DV and in such instances, victims were regarded as the arbiters of their own abuse. This is reflective of other studies that show that norms related to the justification of violence against women are predictive of its prevalence (Heise, 2011). Within focus group discussions, there was little concern expressed for female victims of DV and many men indicated that they had become so desensitized to violence that they did not consider it was a matter of great concern, or they diminished its effects.

## Conclusion

This study is limited in several respects, most notably the fact that the focus groups were not a representative sample of men and young adults, and in including males who had participated in a treatment program, the insights and behavioral changes derived may have influenced the findings in ways that were not reflective of the overall sample. Future research should address these limitations. There is also a need to explore factors that enable men to desist from violence even when they have been socialized in contexts in which DV was normative. A further limitation is that the data from the countries and age groups were merged and it was not possible therefore to investigate country-specific or age-specific differences.

Despite its limitations, the study yields important insights and shows that DV is not only sustained through gender inequalities and intergenerational processes that are given shape within domestic spheres but also through social, cultural, and structural factors in the external environment. Through this coterminous layering of violence reproduction, negative gender attitudes are normalized and behaviors that contribute to DV bolstered. Also, the findings indicate that though rates of violence against women in the Caribbean are said to be among the highest in the world (Jeremiah et al., 2013; 2017), insufficient recognition has been given to violence victimization experienced by males and especially boys and young adults. This is important because it is widely acknowledged that environmental pathogens such as childhood trauma and exposure to violence lead to reduced empathy capacity, lack of guilt, and callous-unemotional traits which inter alia, may be prognostic of future DV (Davis et al., 2018; Debowska, Boduszek, Sherretts, et al., 2018; Debowska, Boduszek, Jones, et al., 2018; Flood & Pease, 2009). The authors contend that at the policy level, there is a need to therefore, to treat DV, other forms of interpersonal violence and violence against children as a conjoint problem with different manifestations rather than simply as different problems with conjoint manifestations. This approach would have cross-cultural benefits in enabling an exploration of the ways in which typologies of violence inform and reproduce each other and the multiple distal and proximal factors that endorse them in whatever context in which they exist. A syncretic framework for violence prevention is called for which recognizes:

1. The intergenerational transmission of negative gender attitudes and violence-affirming behaviors as contributing to its normalization and acceptance.
2. The coterminous layering of violence, meaning that violence within the home is fuelled by factors outside it and vice versa.
3. DV is informed by culturally/historically inscribed patriarchal constructs which are bolstered by the structural location of females in relation to male status and privilege.
4. The need to engage boys, young adults, and men in violence prevention.
